# Droplet-based microfluidics platform for antifungal analysis against filamentous fungi

**DOI:** 10.1038/s41598-021-02350-8

**Published:** 2021-11-26

**Authors:** Sehrish Iftikhar, Aurélie Vigne, Julia Elisa Sepulveda-Diaz

**Affiliations:** Elvesys — Microfluidics Innovation Center, Elvesys, Paris, France

**Keywords:** Biological techniques, Microbiology, Plant sciences

## Abstract

Fungicides are extensively used in agriculture to control fungal pathogens which are responsible for significant economic impact on plant yield and quality. The conventional antifungal screening techniques, such as water agar and 96-well plates, are based on laborious protocols and bulk analysis, restricting the analysis at the single spore level and are time consuming. In this study, we present a droplet-based microfluidic platform that enables antifungal analysis of single spores of filamentous fungus *Alternaria alternata*. A droplet-based viability assay was developed, allowing the germination and hyphal growth of single *A. alternata* spores within droplets. The viability was demonstrated over a period of 24 h and the antifungal screening was achieved using Kunshi/Tezuma as antifungal agent. The efficacy results of the droplet-based antifungal analysis were compared and validated with the results obtained from conventional protocols. The percentage inhibitions assessed by the droplet-based platform were equivalent with those obtained by the other two methods, and the Pearson correlation analysis showed high correlation between the three assays. Taken together, this droplet-based microfluidic platform provides a wide range of potential applications for the analysis of fungicide resistance development as well as combinatorial screening of other antimicrobial agents and even antagonistic fungi.

## Introduction

Plant pathogenic fungi destroy a third of all food crops annually, causing economic loss and impacting global poverty. Among these phytopathogenic fungi is *Alternaria alternata* causing brown spot disease in potato crops. Brown spot, commonly known as “the other early blight”, is an important disease of potato^1^. The symptoms include small circular brown lesions which appear on the abaxial sides of leaves. The disease has the potential to reduce the yield up to 30% and up to 70–80% losses are also reported when the disease is left uncontrolled^2,3^. In storage, *A. alternata* causes black pit on tubers resulting in post-harvest losses of 10%^4^. Several fungicides are used to control the disease, to increase productivity, and to improve the storage life of harvested crops. Intensive use of site-specific fungicides leads to the development of fungicide resistance in *A. alternata*^5,6^ and in various other fungi^7–9^ which necessitates the discovery of new antifungal agents. Discovery and development of new fungicides face great challenges including (a) screening of large libraries of antifungal candidates to evaluate their efficacy, phytotoxicity, and other possible effects, (b) high cost of product development driven by the extensive studies, and (c) complex in vitro and in vivo experiments^10,11^. A new crop protection product takes around 10 years and approximately 260 million USD to be developed (from discovery to first sales)^12^. For antifungal screening, various conventional techniques are used, including agar plates and 96-well plates^13,14^. However, the different methods of analysis may result in different sensitivities, affecting the reliability and usability of test results and thus can influence the choice of the “real hit” as an antifungal agent against a given fungi. The differences in sensitivities may result in biased conclusions from the observed response of the fungicide and depending on the protocol used. Moreover, some of the main limitations of multi-well platforms are the amount of expensive experimental reagents and consumables required, their time-consuming and laborious protocols, and their closed system designs (no flow through reagents or culture medium). The classical approaches are also limiting in terms of single spore analysis and particular difficulties, such as handling and reproducibility can lead to considerable variability between observations within experimental runs. Therefore, it is important to establish a new validated, standardized method for antifungal analysis that can overcome such limitations.

The search to find new molecular targets specifically for antifungal agents is boosted by the advances in microfluidic approaches. For the last two decades, microfluidics has been the focus of interest pertaining to the advantages it presents compared to traditional assays, such as (a) downsizing a bench-top laboratory to a chip, (b) low reagent consumption^15^, (c) high-throughput (HT) analysis^16^, (d) reduced analysis time, (e) monitoring of a high spatial and temporal resolution, (f) observing the dynamic behavior of many cells^17,18^ (g) providing information about cell-to-cell variation in a heterogeneous microbial population,^19,20^ and (h) studying hyphal interactions among fungi^21^. These advantages make microfluidic devices a versatile tool for antifungal screening and analysis. Among different microfluidic approaches, droplet-based microfluidics has emerged as an increasingly interesting alternative to conventional microtiter plate approaches for enzymatic HT screening of fungi^22–24^. These methods can provide an alternative approach for single spore encapsulation^25^ and have the potential to become a powerful tool for large-scale antifungal analysis. Despite its potential for rapid, cost-effective, HT screening, droplet-based microfluidics is not currently used for routine antifungal testing and analysis, most probably given the technical difficulties such as different culture surfaces, reduced media volumes, and vastly different rates of, and methods for, medium exchange that microfluidic tools represent for most experienced plant biologist. Importantly, the encapsulation of a single spore of phytopathogenic fungi into a droplet, considered as a microreactor, provides protection against harsh external environmental conditions, enables the physical and chemical isolation of the spore, and reduces the chances of contamination by foreign organisms^26^. Furthermore, new insights into fungicide molecular mechanisms can be obtained by observation at the single spore level via enhanced dynamic control over the microenvironment by selectively providing nutrients or antifungal agents through the injection of different reagents into the droplet. Nowadays, there are various established techniques for encapsulating a single spore, including layer-by-layer deposition^27^, solvent evaporation^28^, and interfacial polymerization^29^. However, these techniques use various cytotoxic compounds, corrosive chemicals or organic solvents, which jeopardize the viability of the encapsulated organisms^30^. Therefore, a droplet-based approach tackling these challenges and using biocompatible reagents might hold a potential to progressively transition to single spore analysis for antifungal screening for routine assays, which could ultimately revolutionize how fungicides are developed and how fungicide resistance is controlled in fungi.

The main objectives of this study were to establish a simple microfluidic device capable of (a) encapsulating single spores of fungi, (b) performing antifungal analysis, and (c) quantifying gradient-based antifungal-dose response. The platform described herein allows evaluation of the direct effect of antifungal agents on spore germination in a confined microenvironment, with high reproducibility and enabling the quantification of spore germination using widely accessible microscope image analysis. Our design incorporates micron-sized traps that confine the encapsulated spore, allowing their individual visualization and assessment over time by bright-field microscopy. To validate the antifungal assessment results using the microfluidic platform, we also performed assessment experiments with two conventional methods i.e. water agar (WA) and 96-microtiter plate. In the present work, we demonstrate the design and characterization of a droplet-based antifungal analysis platform with precise fluidic control, as a new benchmark for rigorous, reproducible, single spore encapsulation, and antifungal analysis.

## Results

### Disease incidence and disease severity

**Figure 1:**
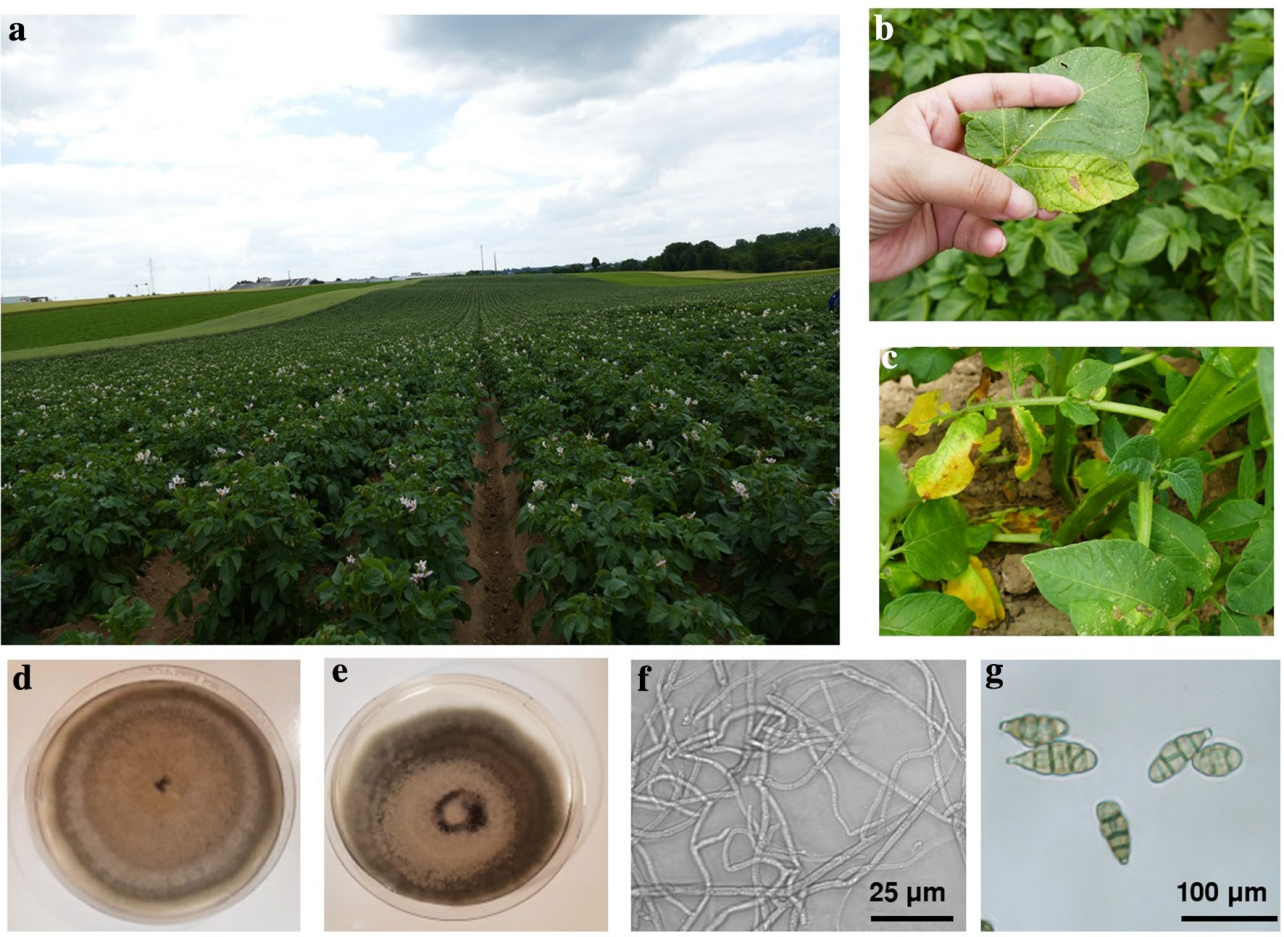
(**a**) Photograph showing the sampling area of a potato field infected with brown spot disease, (**b**, **c**) development of brown spot symptoms on potato leaves with small black to brown lesions and yellowing of leaves, (**d**, **e**) morphological characteristics of *A. alternata* on PDA (**d**) and V8 agar (**e**) after 7 days of incubation at 26 ± 1 °C, (**f**) mycelium of *A. alternata*, (**g**) spores of *A. alternata*.

Sampling was performed from the infected leaves of potato plants showing the symptoms of brown spot disease (Fig. 1a–c). Samples were taken from fifteen different potato varieties namely Accoustic, Allians, Aloutte, AR09-1825, AR10-6283, AR11, Beyonce, Carolus, Ditta, Jelly (organic and fungicide-treated), Levante (organic and fungicide-treated), Monaliza, Monforma (fungicide-treated), Twinner, and Twister (organic and fungicide-treated). The maximum disease incidence was 77.53% in the Twinner followed by 72.41% in Beyonce. Overall, the disease incidence varied from 22.5% in Twinner from the field treated with fungicide to 77.53% in Twinner from the field without any fungicide application. The maximum disease severity was also observed in Twinner 66.9%, followed by 45.74% in variety AR09-1825. Most of the fields were infected by fungus, however, the severity was not very high. Overall, the disease severity ranged from 17.86% in Carolus to 66.9% in Twinner (see Supplementary Table S1 online).

### Isolation and characterization of the fungi on morphological and molecular basis

The isolated fungal species associated with brown spot-affected potato leaves was identified as *Alternaria alternata* on the basis of morphological characters using the taxonomic key of Simmons^31^. The fungus grew well on both conventional culture media, i.e. potato dextrose agar (PDA) and V8 agar, but growth was better on PDA (8.8 cm colony diameter) as compared to V8 agar (8.6 cm colony diameter). The colony color was olive brown on PDA (Fig. 1d) and grayish brown on V8 agar (Fig. 1e). The topography of the colony was spreading, hairy, and velvet-like. At first, the fungus produced dark colored mycelium, ranging from dark brown to gray with hues of brown or olive (Fig. 1f). The sporophores (25–42 μm × 4–24 μm) were septate, straight, and flexuous pale and the spores were olive-brown to pale golden and measured 11–31 μm × 5–12 μm (Fig. 1g). The shape of spores was ovoid or obclavate with one to three longitudinal and two to six transverse septa (Fig. 1g). Based on morphological characteristics of the isolate, the isolate was identified as *Alternaria alternata*.

Taxonomic identification of the strain was performed based on 16S rDNA sequencing. Genetic relationship among *A. alternata* was observed by the neighbor-joining tree assembled on the basis of the ITS1 of *A. alternata* and those available in the database. The neighbor-joining tree revealed that on the basis of ITS1 gene sequence, *A. alternata* (HG995104) showed maximum homology with other sequences of *A. alternata* MW361299, MW361288, MW081304, MW009046, MW009023, MW009021, MW008990, MN822550, MN822543, MN822533, MN822532, MN822530, MN822521, MN822520, MN822513, and KM458821. A comparative analysis of ITS1 gene sequences showed that the fungal strain was very close to *A. alternata* isolates (see Supplementary Fig. S1 online).

### Droplet-based antifungal analysis platform against filamentous fungus *Alternaria alternata*

#### Encapsulation of single spores in a microfluidic droplet

**Figure 2:**
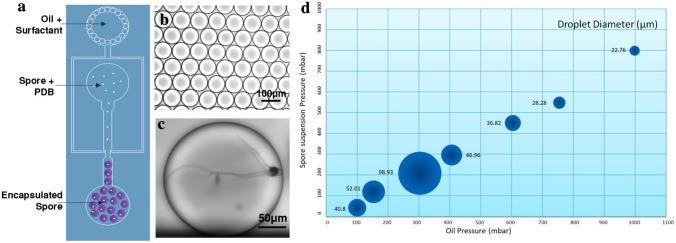
(**a**) Schematic representation of droplet generation chip for single spore encapsulation, (**b**) highly monodisperse droplets (100 µm), (**c**) encapsulated spore of *Alternaria alternata* showing branched hyphal growth after 24 h of incubation inside a droplet, (**d**) correlation of droplet size and pressure for the solutions.

The setup for single spore encapsulation was constructed by bonding a layer of poly-dimethylsiloxane (PDMS), having microchannels embossed into the surface at a depth of 50 μm, to a glass slide. Through bonding of the structured PDMS surface onto a glass slide, the channels and two inlets were created, first for oil (with surfactant) and second for spore suspension (in half strength PDB). Half strength PDB was used to avoid variability in spore germination. The size of the nozzle was 50 μm and the droplets were produced by flow-focusing of the aqueous stream with two streams of fluorinated oil containing surfactant (Fig. 2a) (see Supplementary Movie S1). The designed chip enabled the generation of monodispersed droplets in PDB in oil and encapsulation of single spores of *Alternaria alternata* in PDB (Fig. 2a,b). To set the optimal conditions for single spore encapsulation, various pressures for oil and spore suspension were tested. A droplet size of 100 µm was found to be optimal for the germination of single *A. alternata* spore, while leaving sufficient room for the hyphae to grow after 24 h of incubation (Fig. 4b,c). Encapsulating single spores in droplets allowed growth of the branched mycelial network for up to 24 h confined in the droplet. The growth was observed at 4 h and 24 after encapsulation. The encapsulated spores did not show any abnormalities in germination, cell wall development, or hyphal growth (Fig. 2c). The droplets were collected off-chip in a vial filled with oil and surfactant. The droplet size coefficient variation (CV) was of 1% (i.e. monodisperse droplets). CV is the coefficient variation that expresses the monodispersity or polydispersity of the droplets. Of note, size and frequency of the droplets are usually dependent on the pressure, flow rate, and viscosity of the liquids used. The resulting sizes of droplets as a function of pressures applied to both liquids are shown in Fig. 2d.

#### Microfluidic platform for on-chip antifungal analysis

**Figure 3:**
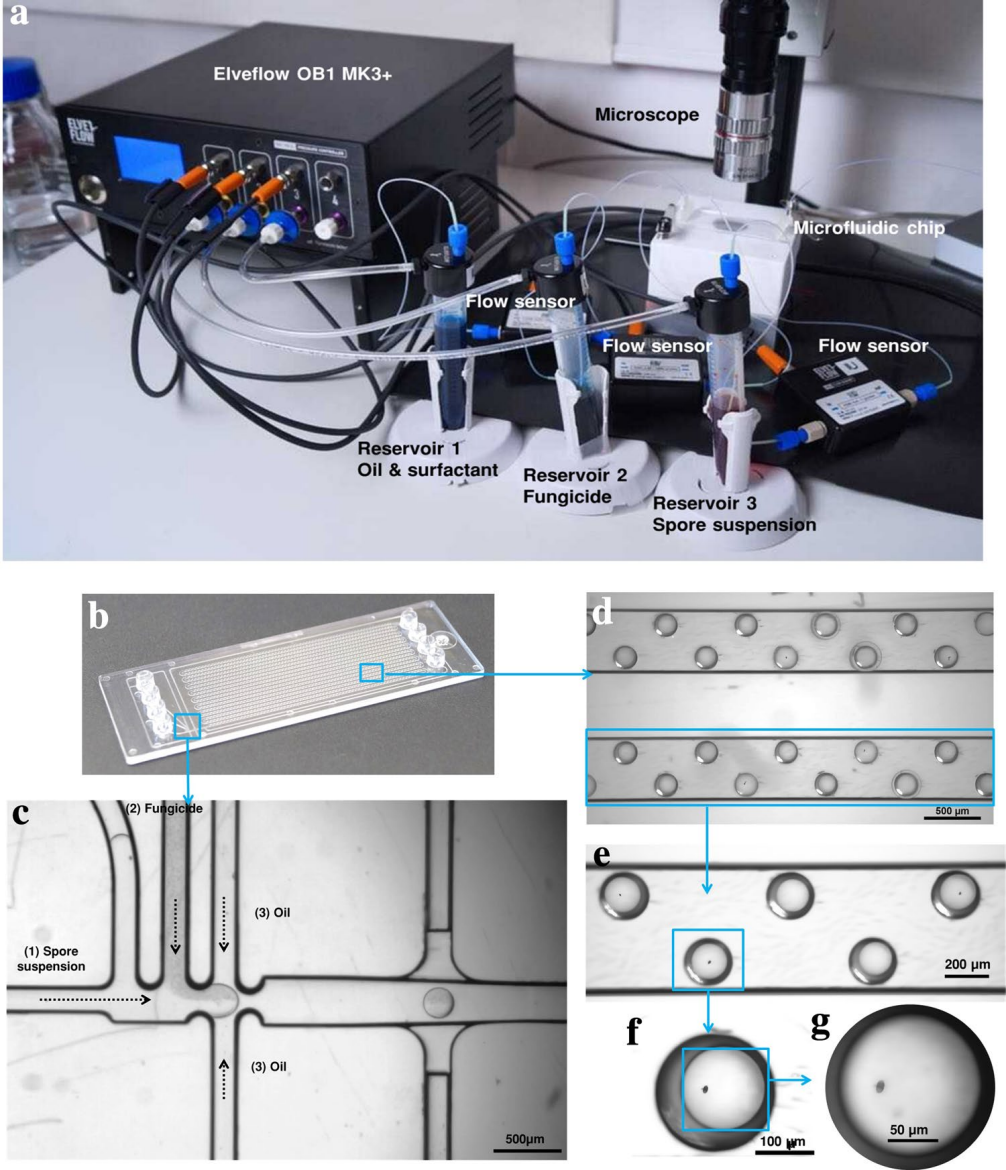
(**a**) An overview of the droplet-based microfluidic platform used for single spore encapsulation and antifungal analysis, (**b**) overview of PC microfluidic chip (Fluidic 719, Microfluidic ChipShop, Germany), (**c**) droplet generation process using three inlets (1) spore suspension inlet, (2) fungicide inlet and (3) oil inlet, (d-g) droplets trapped inside the storage positions from 500 to 50 µm magnifications.

The antifungal analysis platform included an OB1 flow controller (Elveflow, France), tubings, connectors, 50-mL polystyrene tubes, a polycarbonate (PC) microfluidic chip (hydrophobic channels), and an optical microscope for observation (Fig. 3a). The OB1 pressure controller is used to reliably generate monodisperse droplets (CV < 3%) at a high-throughput. Due to the possible absorption of small hydrophobic molecules like spore-derived biomolecules and drugs by PDMS, these chips used to perform viability assays of the spores in the system and to optimize droplet size for encapsulation. For the final microfluidic analysis platform, a chip made of PC (Fluidic 719, Microfluidic ChipShop, Germany) was used (Fig. 3b). The commercially-available chip is composed of two distinct sections: (1) droplet maker and (2) storage positions for droplets (2261 in total). A drop maker (nozzle size 82 µm) allowed the encapsulation of single spores with specific concentration of fungicide (Fig. 3c) (see Supplementary Movie S2 online). The architecture of the microfluidic chip is designed to generate droplets by hydrodynamic flow focusing by merging two streams of aqueous fluids: one carrying a suspension of spores and the other carrying a specific concentration of fungicide. The media PDB containing the suspension of spores and the antifungal agent (kunshi) were each pumped at 200 mbar, while HFE7500 fluorinated oil + 1% v/v surfactant was pumped at 210 mbar, with fine-tuned pressures allowing for a 1:1 mix of aqueous fluids. The resulting droplets of 100 µm size were trapped in specific storage positions on the chip for 24 h incubation (Fig. 3d–g) (see Supplementary Movie S3 online). The traps allowed the visualization of each spore for the observations at 4 h and 24 h of incubation. The throughput of droplets was 5.4 × 10^3^ droplets (diameter of 100 µm) produced per hour (oil pressure = 210 mbar, spore suspension pressure = 200 mbar, fungicide pressure = 200 mbar). The mean number of spores per droplet (λ) was 0.01, and the single-spore droplet was 70 (n = 12) with occupancy of 0.03%, according to the law of Poisson distribution. The number of droplets with two spores was 8 (n = 12) with occupancy of 0.003%. The number of droplets with no spores was 2183 (n = 12) with occupancy of 0.965% empty droplets. The droplet size was calculated at different times points, at t = 0 h (100 µm), t = 4 h (99.97 µm) and t = 24 h (99.47 µm). During the experiments, optimal growth conditions were maintained within the droplet by providing the media and a sterile environment.

**Figure 4:**
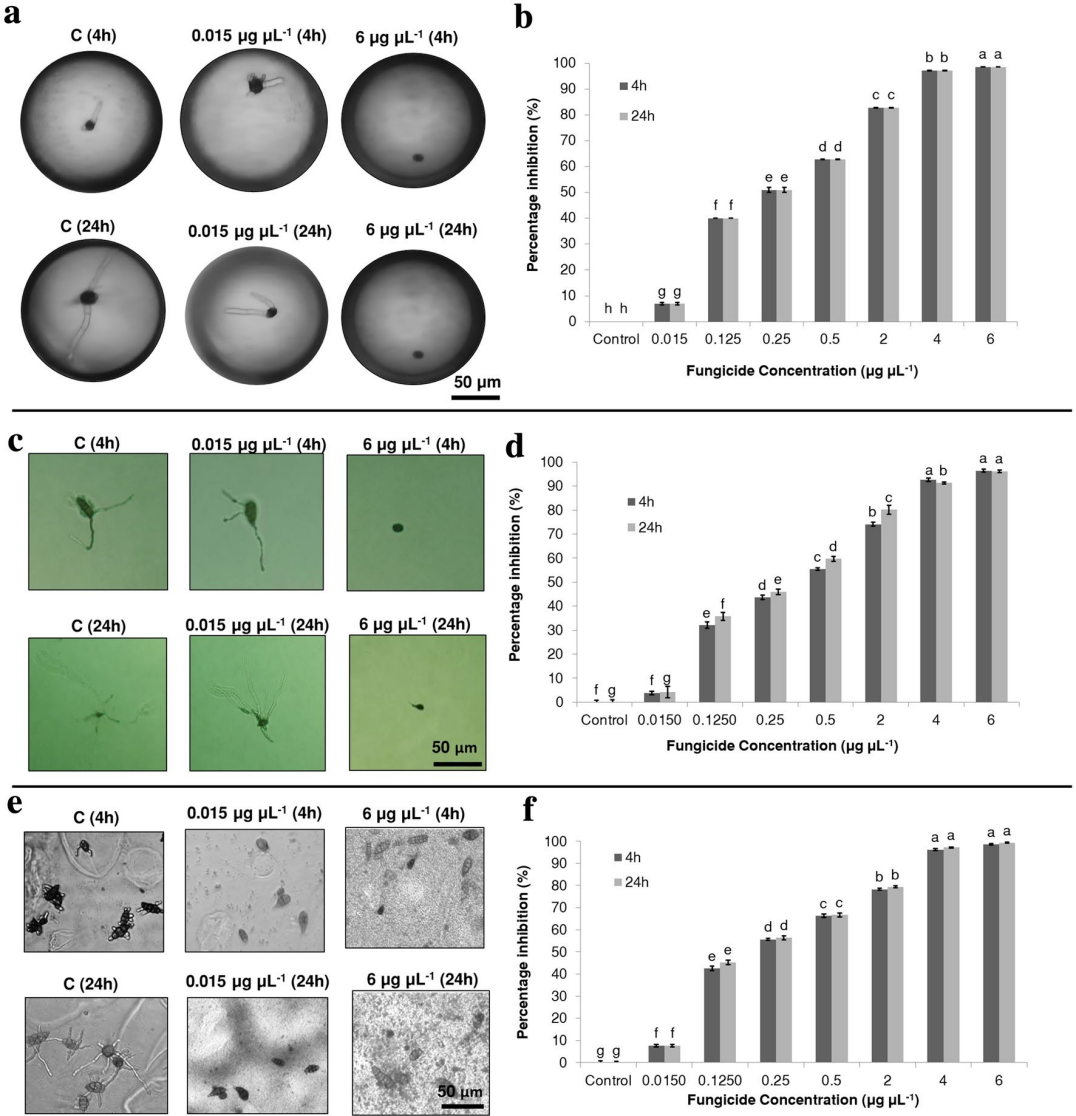
In vitro antifungal efficacy of kunshi against spore germination of *Alternaria alternata* assessed by droplet-, WA, and 96-well plate assays. (**a**) spores of *A. alternata* after treatment with fungicide at 4 h and 24 h incubation assessed by droplet assay, scale bar 50 µm (**b**) percentage inhibition of kunshi against *A. alternata* in droplet assay, (**c**) spores of *A. alternata* after treatment with fungicide at 4 h and 24 h incubation assessed by WA assay, scale bar 50 µm, (**d**) percentage inhibition of kunshi against *A. alternata* in WA assay, (**e**) spores of *A. alternata* after treatment with fungicide at 4 h and 24 h incubation assessed by 96-well plate assay, scale bar 50 µm, (**f**) percentage inhibition of kunshi against *A. alternata* in 96-well plate assay. Error bars represent ± standard error of means (n = 6 in case of WA and 96-well plate, n = 140 in case of droplet assay). Each concentration with different letters is significantly different from each other (*P *< 0.05; Statistix 8.1). The significance was calculated by Tukey's HSD post-hoc test (*P* = 0.05).

The platform was used to assess the antifungal efficacy of kunshi against spore germination of *Alternaria alternata *via encapsulating the single spore in droplets with specific concentration of kunshi. A total seven different concentrations of kunshi ranging from 0.015 to 6 µg µL^−1^ were used. The dose of 6 µg µL^−1^ showed a maximum of 98.6% reduction in germination after 4 h and 24 h incubation which was significantly (*P *< 0.05) higher than the control. The least reduction of spore germination (6.9%) was obtained at the concentration of 0.015 µg µL^−1^ which was significantly (*P *< 0.05) higher than the control. All the concentrations showed significantly (*P *< 0.05) higher inhibition percentage as compared to control at both the time points (4 h and 24 h after incubation) (Fig. 4a,b). Also, the percentage inhibition between 4 and 24 h time points for each concentration was not significantly different (*P* < 0.05) from each other.

The antifungal activities of kunshi were expressed as EC50 (median inhibitory concentrations) against spore germination of *A. alternata* after 4 h and 24 h incubation with the fungicide. Data revealed that kunshi displayed dose-dependent antifungal activities against spore germination of *Alternaria alternata*. Overall EC50 values ranged from 0.125 to 0.25 μg μL^−1^ at both the time points 4 h and 24 h (Table 1).

**Table 1:** Mean EC50 values (effective concentration that inhibited spore germination by 50%) for *Alternaria alternata* obtained from in vitro assessment of kunshi after 4 h and 24 h of incubation at 26 ± 1 °C.

Method	EC50 (µg µL^−1^)	
	4 h	24 h
Droplet-based assay	0.125–0.25	0.125–0.25
Conventional agar plate assay	0.25–0.5	0.25–0.5
Microtiter plate assay	0.125–0.25	0.125–0.25

### Conventional agar plate assay

Kunshi was screened against *Alternaria alternata *via an in vitro water agar (WA) spore germination assay to evaluate its antifungal efficacy. In total, seven different concentrations ranging from 0.015 to 6 µg µL^−1^ of commercially available fungicide kunshi were used. The dose of 6 µg µL^−1^ showed a maximum of 96.1% and 96.3% reduction in germination after 4 h and 24 h incubation, respectively, which was significantly (*P* < 0.05) different from the control. The least reduction of spore germination was obtained at the concentration of 0.015 µg µL^−1^ which was not significantly (*P* > 0.05) different from the control. All concentrations, except above 0.015 µg µL^−1^, showed significantly (*P* < 0.05) higher inhibition percentage as compared to control after 4 h and 24 h of incubation. Data also revealed that overall kunshi displayed dose-dependent antifungal activities against *Alternaria alternata* (Fig. 4c,d). Overall EC50 values ranged from 0.25 to 0.5 μg μL^−1^ at both time points 4 h and 24 h after incubation (Table 1).

### Conventional microtiter plate assay

Kunshi was also tested in 96-well plate assay to screen its antifungal effect on *Alternaria alternata* spore germination. Kunshi (6 μg μL^−1^) showed a maximum of 98.5% and 99.3% reduction in germination after 4 h and 24 h incubation, respectively, which was significantly (*P* < 0.05) different from the control.. The least reduction of spore germination was obtained by 0.015 μg μL^−1^ which was 7.63% and 7.64%, after 4 h and 24 h of incubation, respectively, significantly (*P *< 0.05) different from the control. All the concentrations displayed significantly (*P *< 0.05) higher inhibition potency when compared to the control (Fig. 4e,f). Overall EC50 values ranged from 0.125 to 0.25 μg μL^−1^ at both the time points i.e. 4 h and 24 h of incubation (Table 1). The compounds displayed dose-dependent antifungal activities.

### Validation of antifungal efficacy results assessed by droplet-based analysis

The efficiency of the platform for antifungal assessment was validated by comparing the results of antifungal potential of kunshi assessed by droplet-based analysis and by WA and 96-well plate assays (Table 2). Percent inhibition was lowest using the WA assay. At all the tested concentrations, the percentage inhibitions assessed by 96-well plate and droplet assay were closer to each other than to those assessed by WA (Table 2) after 4 h incubation. At both 4 h and 24 h time points, the percentage inhibition calculated using the droplet assay fell between values obtained by WA and 96-well plate assays for all drug concentrations ≤ 0.5 µg µL^−1^, while at concentrations ≥ 2 µg µL^−1^, the highest inhibition was recorded in the droplet assay (Table 2). The viability of the fungal spores in all the three assays (water agar, 96 well plate and droplet assay) without the use of antifungal agents was 100%.

**Table 2:** Comparison of in vitro inhibition potential of kunshi against spore germination of *A. alternata* assessed via three assays i.e., WA, 96-well plate, and droplet assay.

Time	Assays	Concentrations (µg µL^−1^)							
		0	0.015	0.125	0.25	0.5	2	4	6
		Percentage inhibition (%)							
4 h	Droplet	0 ± 0 g	6.9 ± 0.4 f.	40 ± 0 e	51.7 ± 1 d	62.7 ± 0.9 c	82.8 ± 0.09 b	97.2 ± 0.09 a	98.6 ± 0.04 a
	WA	0 ± 0.7 h	3.8 ± 0.7 g	32 ± 1.3 f	43.6 ± 0.8 e	55.4 ± 0.4 d	74.0 ± 0.8 c	92.5 ± 0.7 b	96.3 ± 0.6 a
	96-wellplate	0 ± 0.7 h	7.6 ± 0.5 g	42.5 ± 1 f	55.6 ± 0.4 e	66.3 ± 0.6 d	78.2 ± 0.3 c	96.1 ± 0.4 b	98.5 ± 0.3 a
24 h	Droplet	0 ± 0 g	6.9 ± 0.4 f.	40 ± 0 e	51 ± 1 d	62.7 ± 0.09 c	82.8 ± 0.09 b	97.2 ± 0.09 a	98.6 ± 0.04 a
	WA	0 ± 1 f	4.1 ± 2 f.	35.7 ± 1.6 e	46 ± 1.1 d	59.7 ± 1 c	80.1 ± 1.9 b	91.3 ± 0.4 a	96.1 ± 0.4 a
	96-wellplate	0 ± 0.5 h	7.6 ± 0.5 g	45.3 ± 0.9 f	56.4 ± 0.8 e	66.6 ± 0.8 d	79.3 ± 0.4 c	97.2 ± 0.2 b	99.3 ± 0.2 a

Each concentration with different letters is significantly different from each other (*P *< 0.05; Statistix 8.1). The significance was calculated by Tukey's HSD post-hoc test (*P* = 0.05). ± Represents standard error of means (n = 6 in case of WA and 96-well plate, n = 140 in case of droplet assay).

The relationship among different methods of antifungal analysis was further investigated using Pearson correlation analysis (Table 3). The correlation analysis between droplet assay and 96-well plate assay showed significantly high positive correlation (r = 0.9974, *P *< 0.05) after 4 h of incubation. The antifungal efficacy in droplet assay was positively correlated with efficacy in 96-well plate assay (r = 0.9964, *P *< 0.05). A positive correlation (r = 0.9990, *P *< 0.05) was also found between droplet and WA assay after 24 h of incubation (Table 3).

**Table 3:** Pearson correlation among three different antifungal assessment assays against *A. alternata* after 4 h and 24 h of incubation.

	Assay	Value	WA	96-well plate
4 h	96-well plate	r	0.9917	
		p	< 0.05	
	Droplet assay	r	0.9969	0.9974
		p	< 0.05	< 0.05
24 h	96-well plate	r	0.9929	
		P	< 0.05	
	Droplet assay	r	0.9990	0.9964
		p	< 0.05	< 0.05

Correlation (r) is significant at *P *< 0.05; Statistix 8.

The antifungal activities measured by droplet assay correlate with those from microtitre plates and WA assays. These results validate that the antifungal analysis performed via droplet assays fell between the results assessed via WA and 96-well plate assays. Altogether, the platform is versatile in 3 major ways: (1) on-chip encapsulated spores can be incubated and analyzed at different time points and up to 24 h, (2) encapsulated single spores in droplets allowed growth of the branched mycelial network for up to 24 h confined in the droplet, and (3) dose-dependent antifungal analysis can be performed in encapsulated spores.

## Discussion

In the present study, we have successfully developed a droplet-based microfluidic platform to perform antifungal analysis on single encapsulated spores of phytopathogenic fungus, *Alternaria alternata*. This work relied on a series of key novel advances in the field of plant pathology and fungicide analysis methods, for instance: (1) the development of an on-chip method for single spore encapsulation, (2) the demonstration of a biocompatible droplet-based assay using *A. alternata* spore as a model system of filamentous fungi that survives up to 24 h inside a droplet, and (3) the ability to perform antifungal analysis using a microfluidics droplet-based system. The microfluidic-based fungicide analysis platform allowed reproducible antifungal analysis. Previously, research has been conducted specifically to perform droplet-based manipulations, such as cell encapsulation^32–34^, droplet mixing^35^, on-chip incubation^36–38^, droplet merging^39,40^, and droplet sorting^41,42^. However, each of these droplet manipulations has been demonstrated as separate modules that have not been assembled into an integrated antifungal analysis assay. Here, by combining an on-chip spore viability and antifungal analysis assays in droplets, we were able to develop a droplet-based platform for conducting fungicide analysis against plant pathogenic filamentous fungus *A*. *alternata*.

To validate the platform for antifungal analysis, similar experiments were performed using WA and 96-well plate assays. For all the three assays, the percentage inhibition of spore germination of *A. alternata* was measured at two time points, i.e. after 4 h and 24 h of incubation to check the persistence of the antifungal efficacy of kunshi. At all the tested concentrations, the percentage inhibitions assessed by droplet assay were closer to 96-well plate and WA assays (Table 2). These results were further confirmed by Pearson correlation analysis (Table 3). Pearson's correlation coefficient measures the statistical relationship, or association, between two continuous variables. It is known as the best method of measuring the association between variables of interest because it is based on the method of covariance. The analysis showed that a high correlation was observed between 96-well plate assay and droplet assay at 4 h of incubation (Table 3). This high correlation between microtiter and droplet assay can be explained as an environment saturated with PDB, resembling the availability of water and nutrients, highly similar to that in a conventional 96-well plate. A correlation analysis revealed that the results obtained on the platform are not significantly (*P *< 0.05) different from those obtained in WA and 96-well plate at 24 h. Hence, the platform can be used as a tool to perform antifungal analysis, which would yield results comparable to those obtained with the conventional 96-well plate and WA assays. The lower correlation between the droplet assay and WA found at 4 h of incubation can be explained by the fact that WA techniques depend on diffusion of the test compound into the medium and thus the concentration within the medium is not homogeneous in contrast to the dilution methods. The overall differences in inhibition percentages among droplet assay and 96-well plate and WA assays are due to the analysis of the spores in bulk for the WA and 96-well plate methods, an issue tackled by the droplet-based platform through the direct visualization of the encapsulated spores in the microfluidic chip. Moreover, the microfluidic chip has storage positions enabling the observation of the same spore independent of the time of analysis, presenting an advantage over the 96-well plate and WA assays. This asset is one of the main features of the droplet-based platform. Finally, in the three different spore germination assays, the spore inhibition with half maximal effective concentration (EC50) was calculated. It ranged from 0.125 to 0.25 μg μL^−1^ evaluated via droplet- and 96-well plate assays, while the EC50 ranged from 0.25 to 0.5 μg μL^−1^ evaluated via WA assay, showing that the compounds exhibited dose-dependent antifungal activity.

There have been relatively a few published reports in which the methods for determining antifungal efficacy against plant pathogens have been compared. To our knowledge there have not been significant advances in methods for fungicides assessment assays^43,44^. In observations of techniques involving spore germination, Gottlieb^45^ found the seeded, toxicant-agar method, and the test tube dilution method were equally sensitive. These results are in accordance with the present results where percentage inhibition measured by all the three assays are close to each other and are also closely correlated to each other showing that the droplet-based analysis can yield the equally sensitive results. In another study, the oils of bay, cinnamon leaf, clove, lemongrass, mustard, orange, sage, thyme, and two rosemary oils were tested by two methods: (1) a rye bread-based agar medium supplemented with 100 and 250 μL L^−1^ essential oil and (2) real rye bread exposed to 136 and 272 μL L^−1^ volatile oil in air. The results showed that antifungal effects of the essential oils depended on the application method^46^. The comparisons of studies that use different methodologies are difficult and the need for uniform and reliable procedures when testing activity has been emphasized^47^.

The incorporation of a novel droplet-based analysis platform helped to customize and optimize the antifungal assessment techniques that are currently missing from existing in vitro models, providing a platform to study the biology and physiology of the fungal spore. The droplets (diameter of 100 µm) were produced at a throughput of 5.4 × 10^3^ droplets per hour with oil pressure = 210 mb, spore suspension pressure = 200, fungicide pressure = 200. The total throughput of the platform can be enhanced by adding parallel channels, multiple microfluidic chips, and increasing the overall pressure. HT analysis can be achieved by coupling the platform with automatized image analysis of the chip and the traps (using for example automated microscopy and image analysis or high-content screening microscopy approaches). The developed platform can be expandable to other filamentous fungi by optimizing the conditions, depending on the filamentous fungi used, in terms of droplet size, incubation conditions (temperature, photoperiod, media etc.). The droplet based antifungal analysis can be used a standardized method because (a) it uses mainly commercially available instruments that already meet industrial quality levels and production standards, i.e. optical microscope, flow control instruments, thermoplastic chip, (b) some key steps can be semi or fully automated and finely controlled, such as flow rate or volume control. Automation brought by the microfluidic controllers can help to ensure high reproducibility per individual droplet (as an equivalent of a plate well) and reduce the impact of human error, (c) it relies on single spore analysis instead of bulk analysis. The direct visualization of the single encapsulated spores in the microfluidic chip ensures reliability and reproducibility of results thus minimizing the biased conclusions. This also offers the possibility of verifying the potential heterogeneity of the response towards a given fungicide. The specific storage positions in microfluidic chip enable the observation of the same spore independent of the time of analysis, presenting an advantage over the 96-well plate and WA assays, (d) the results replicate the observations made with two conventional methods, thus validating the present system and offering complementary advantages compared to such traditional methods, (e) it includes the bright field microscopy analysis which is the simplest of all the optical microscopy illumination techniques. Simplicity of platform and its integration with basic equipment (light compound microscope) can help adopt the platform as a standard tool for analysis and thus easily replicated by many researchers, (f) the system comprises a commercially available microfluidic chip which already meets large scale standards and can be used as an off-the shelf consumable. Contrary to other microfluidic methods using home-made chips (e.g. PDMS chips, micromilled, etc.) that might bear some variability during the manufacturing procedure, large scale chip production using well-known thermoplastics (e.g. polystyrene, polycarbonate, polymethyl methacrylate, …) commonly fabricated by mould injection usually bear significantly less variability among units and batches, (g) the system is able to perform antifungal analysis with lower infrastructure, labor, and consumables. The confinement of the single spore allows fast tracking and observations within the droplets which can decrease the overall time of assessment. The technique allows fast analysis of antifungal activity and can be integrated to industrial fungicide screening processes to speed up the discovery of new antifungal agents. This advantage will increase the interest for adopting the technique and its progression towards a standardized model. This technique can be adapted to encapsulate different types of fungus, with different spore size (for example, *A. tenuissima*, *Curvularia spp., Fusarium spp*. etc.), or even eukaryotic or prokaryotic cells, e.g. bacterial or mammalian cells. Precise spore encapsulation, HT droplet generation and manipulation, and the possibility of combinatorial screening (by combining 2 antifungal agents for instance) suggest that droplet technology can be used as an ideal platform for a range of screening approaches. We have developed a droplet technology that can be used not only in the context of any large-scale antifungal analysis studies, but is also well-suited to analyze the mode of action of antifungal agents and resistance-development in fungi. Although we only present the data obtained for germination inhibition of *Alternaria alternata*, this platform can be further extended for analysis of the molecular mechanisms of antifungal agents which play a crucial role in cellular mechanisms. A deeper understanding of the mode of action of antifungal agents will help to optimize their application, which in turn contributes to their successful use in food and feed production. The evaporation and leakage from the droplets was minimized by filling the chip with oil right after the generation of droplets. The surfactant (1%) (Emulseo, France) was used which is known to be very effective in limiting the leakage and can efficiently contain the hydrophilic and hydrophobic molecules within droplets. The droplet size at t = 0 h (100 µm), t = 4 h (99.97 µm) and t = 24 h (99.47 µm). The differences in size of the droplets calculated at different time points (t = 0, t = 4 h, and t = 24 h) are negligible which further confirms that the leakage of the antifungal agent in the droplet is insignificant. However, depending on the bioactive molecule used (hydrophobic/hydrophilic), different methods might need additional controls to optimize the leakage from the droplets. In conclusion, we have successfully developed a versatile and efficient microfluidic system for encapsulation of single spores of fungi, followed by antifungal analysis in droplets.

## Materials and methods

### Sampling

A survey was carried out in a potato fields of Desmazières SA, AngriCo Company in Arras, France, to collect the diseased samples of potato from a total of fifteen different varieties including plants treated and untreated with fungicides. An appropriate permission was obtained to collect and process the plant samples. The present study complies with the local and national regulations. The leaf samples with typical brown spot symptoms were excised from petiole, packed, and shipped to the laboratory for the procurement of causal fungus (see Supplementary Fig. S2 online). The percent disease incidence was worked out as per the formula given by^48^. Plant disease incidence (%) represents the number of plants that are visibly diseased relative to the total number of plants assessed. For assessment of percent disease intensity (PDI) (%), the diseased leaves were categorized as per the scale given by^49^ and PDI was calculated using the formula given by^50^. PDI can be defined as the area of plant tissue that is visibly diseased relative to the total plant tissue.

### Isolation and characterization of fungus on morphological and molecular basis

The fungus associated with diseased leaves was isolated. The leaf tissues were cut into small pieces (5 × 5 mm) excised from the edge of lesions and surface sterilized with 1% sodium hypochlorite (NaOCl) for a minute, subsequently washed with sterilized distilled water, and embedded in potato dextrose agar (PDA; Dutscher) (20 g potato dextrose and 15 g agar L^−1^ sterilized H_2_O). The inoculated plates were incubated at 26 ± 1 °C for 3–4 days with 12 h photoperiod. The pathogen was purified by recurrent sub-culturing using the hyphal tip method^51^. The hyphal tips growing out from the tissue sections were cut off and aseptically transferred to PDA plates. Once the colonies were mature, the spore suspension of purified culture was prepared and single spore culture was obtained by inoculating the plates with a few drops of spore suspension. The single spore colonies were allowed to mature on media at 26 ± 1 °C for 7 days and morphological traits of the colony and sporulation structures were determined by a bright-field microscopy using a light compound microscope equipped with an Axicon 202 mono camera (Microscope statif Axio Vert.A1 TL/RL, Carl Zeiss AG, ZEISS, Germany).The radial growth of fungal isolates was studied on two different media namely PDA and V8 (Les jardins de Rully, France) agar. The stock cultures were preserved at 4 °C on PDA plates until further use. The internal transcribed spacer (ITS1 and ITS2) regions were used to characterize the pathogen on a molecular basis. DNA (50–100 ng) was used to generate amplicons using ITS1 and ITS2 primers and sequenced by GENEWIZ (GENEWIZ, Inc., South Plainfield, NJ, USA). The homologous sequences of ITS1 of the similar fungal species were taken from NCBI GenBank using the Basic Local Alignment Search Tool (BLAST)^52^. The alignment of consensus sequences and phylogenetic analysis were performed using the neighbor-joining method with MEGA6 version 6.0^53^.

### Droplet-based antifungal analysis platform

#### Microfluidic PDMS chip fabrication and single spore encapsulation

**Figure 5:**
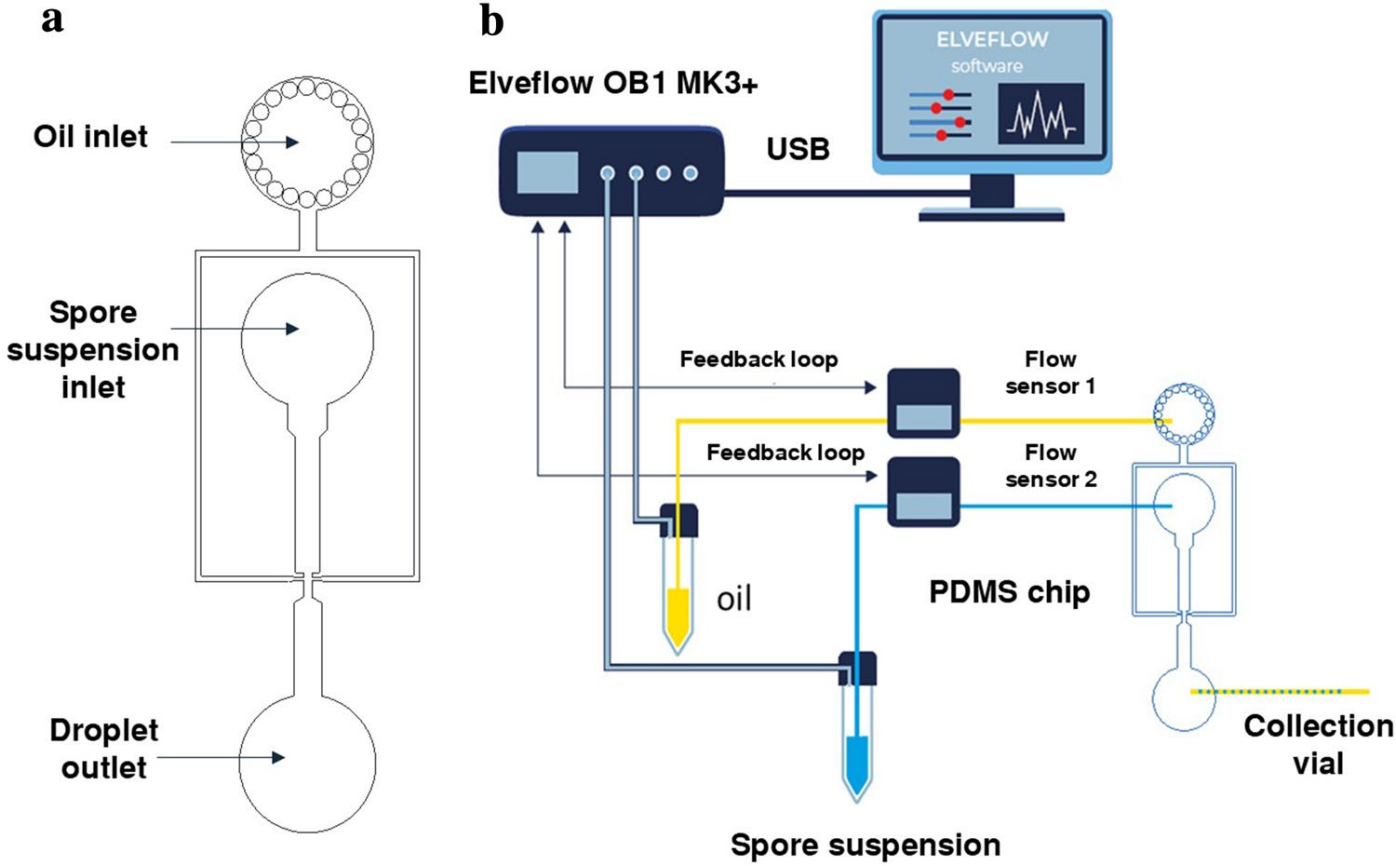
(**a**) Schematic of single spore encapsulation chip: droplets are produced by flow-focusing of the aqueous stream with two streams of fluorinated oil containing surfactant, (**b**) schematic of setup showing the components for single spore encapsulation.

A microfluidic chip was created by soft-lithography technique. The chip was designed under the software AutoCAD 2018 (Autodesk, Inc) (Fig. 5a) and printed on a photomask by CAD/Art (CAD/Art Services, Inc, USA). A mold of SU8-2050 negative photoresist (MicroChem Corp, USA.) was prepared on a silicon wafer (Siltronix) by UV exposure (UV-KUB, Kloé, France) through a photomask and subsequent development (SU-8 developer; MicroChem Corp.). A curing agent was added to PDMS base (Sylgard 184 elastomer kit; Dow Corning Corp.) to a final concentration of 10% (w/w), mixed, and poured onto the mold. After degassing under vacuum, the mold was incubated for five hours at 65 °C. PDMS was then peeled off and inlets and outlets were punched with biopsy punch (Ø 0.75 mm; Eloise, France). The structured side of the PDMS was bound to a glass microscope slide (Corning) by exposing both parts to an oxygen plasma (Harrick Plasma) equipped with Equinox (BlackHole Lab, France). The microfluidic chips were treated with Aquapel (Autoserv, Germany) followed by HFE-7500 oil (Novec7500, 3 M). A spore suspension of a precise concentration (2 × 10^4^ spores mL^−1^) according to Poisson Law^54^ (λ = 0.01) was prepared from 7–8 days old sporulating cultures of *A. alternata* in half strength PDB (Dutscher, France). The spore suspension was filtered using nylon filtration tissue NITEX, mesh opening 50 µm (Dutscher, France) and 0.1% Tween 20 (Sigma, Aldrich) was added. The reservoirs were filled with spore suspension and HFE-7500 fluorinated oil diluted with 1% surfactant (Emulseo, France) and connected to the microfluidic chip by OD 1/32″ tubing. The reservoirs were connected to the OB1 pressure controller (Elveflow, France) outlet to control flow rates through the ESI software (Fig. 5b). The droplet size for single spore encapsulation was optimized by applying different pressures to accommodate single spore.

#### Development of microfluidic platform for on-chip antifungal analysis

**Figure 6:**
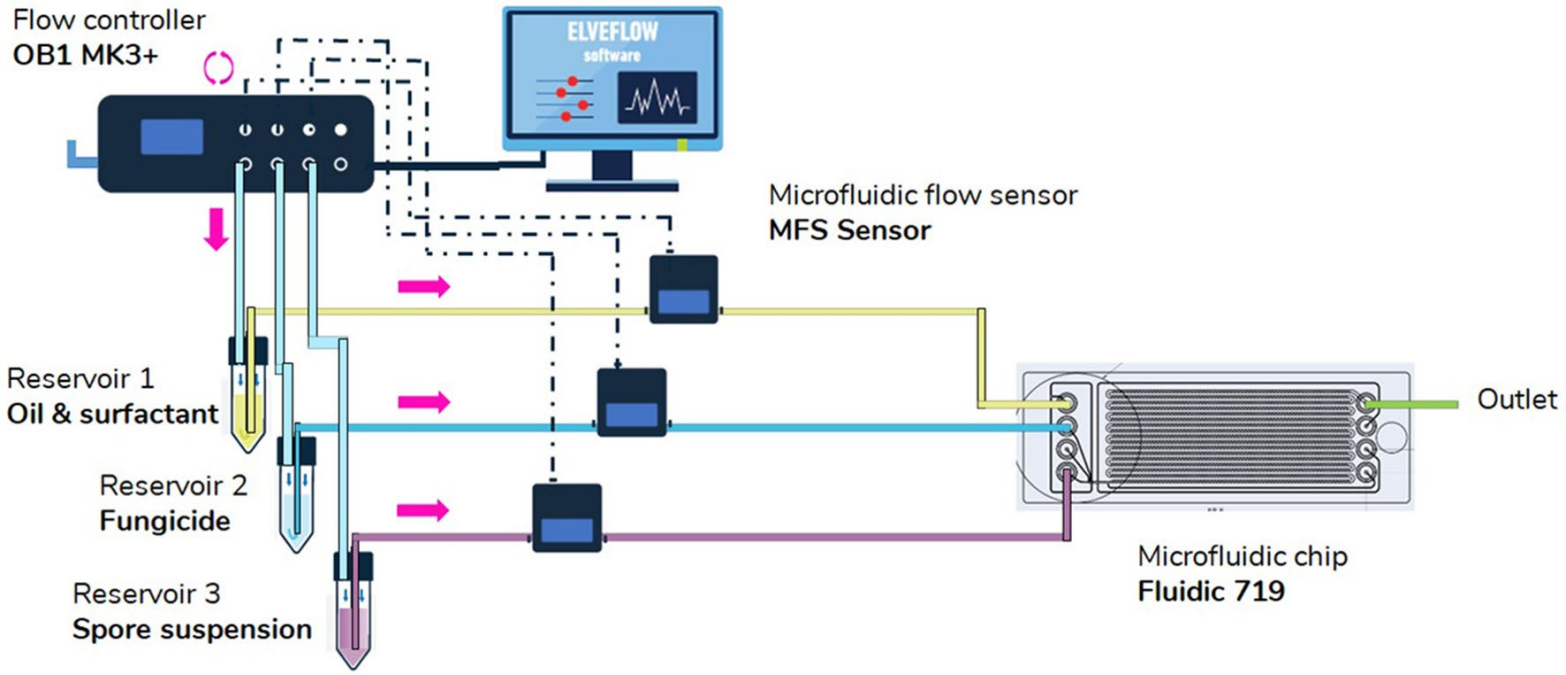
Schematic presentation of droplet-based platform for on-chip antifungal analysis.

A polycarbonate chip (Fluidic 719, Microfluidic Chipshop, Germany) composed of three inlets was used: (1) first inlet for spore suspension, (2) second for fungicide, and (3) third for oil solution (Fig. 3c). The chip combined mini Luer interfaces, nozzle size of 82 µm, T + flow focusing crossing and 2261 storage positions (Fig. 3d). The microchannels were treated and the spore suspension prepared as described previously in “Microfluidic PDMS chip fabrication and single spore encapsulation” section. The commercially available fungicide, kunshi/tezuma (composition: 375 g kg^−1^ fluazinam + 250 g kg^−1^ cymoxanil), provided by Belchim Crop Protection, France, was used in its commercial form to prepare a stock solution (200 μg mL^−1^) in dimethylsulfoxide (DMSO) (1%). Seven different concentrations of kunshi (6, 4, 2, 0.5, 0.25, 0.125, and 0.015 μg μL^−1^) were used in all the assays and DMSO (0.1%) was used as a control. Droplets containing a single spore and specific concentration of kunshi (6–0.015 μg μL^−1^) were trapped in the storage positions followed by on-chip incubation at 26 ± 1 °C for 4 h under continuous light and for 24 h with a photoperiod of 12 h. The schematic presentation of platform is show in Fig. 6. Seventy encapsulated spores per experiment were examined for the development of the germ tube at 40× magnification by bright-field microscopy using a light compound microscope. The spore was considered germinated when the germ tube was equal or longer than the spore, or if multiple germ tubes were emerging from one spore. Inhibition percentage (%) of germination was calculated using the formula by^55^. The effective dose for 50% of spore germination inhibition (EC50) value was obtained by linear regression of Probit analysis^56^. EC50 refers to the concentration of an antifungal agent which induces a response halfway between the baseline and maximum after a specified exposure time. It is commonly used as a measure of a drug's potency. The experiment was repeated twice.

### Conventional agar plate assay

The antifungal potential of kunshi was evaluated via spore germination assays by comparing the spore germination on WA plates amended or not with the fungicide^57^. The aliquots of kunshi were added to WA cooled to 55 °C to obtain desired concentrations (6–0.015 μg μL^−1^). The control was amended with DMSO having a final concentration of 0.1% (v/v). 50 µL aliquots of the spore suspensions of *A. alternata* (1 × 10^5^ spores mL^−1^) were spread on fungicide-amended and fungicide-free Petri dishes (Ø 9 cm) with a glass rod. The plates were incubated at 26 ± 1 °C for 4 h and 24 h. Following incubation (4 h and 24 h), hundred spores per plate were examined for the development of the germ tube by bright-field microscopy using a light compound microscope. The percentage inhibition and EC50 were calculated as described in “Development of microfluidic platform for on-chip antifungal analysis” section. Three replicates were used for each treatment and the experiment was repeated twice.

### Broth microdilution plate assay

10 µL aliquots of spore suspensions of *A. alternata* (1 × 10^5^ spores mL^−1^) were added in each well. Seven different concentrations of kunshi (6–0.015 μg μL^−1^) were used. The total volume of each well was adjusted to 100 μL with half strength PDB. The plates were incubated at 26 ± 1 °C and germination of spores was assessed by calculating the inhibition percentage and EC50 as described previously in “Development of microfluidic platform for on-chip antifungal analysis” section. Three replicates were used for each treatment and the experiment was repeated twice.

#### Image analysis

Images were processed and analyzed using GIMP 2.10.20^58^. The horizontal and vertical diameters of hundred droplets were measured for each experiment (in pixels). The measurements were subsequently averaged (n = 100) and converted into micrometers (1 px = 0.93 μm). For WA and 96-well plate assays, the spores were designated as germinated or un-germinated by direct observation using a light compound microscope.

#### Statistical analysis

All experiments were reproduced as indicated in the methods above. Data were arranged in Microsoft Excel and analyzed in Statistix 8.1^59^. Statistical analyses were performed using data of six independent replicates (n = 6) from two experiment for WA and 96-well plate assays and of seventy single spore encapsulated droplets considered as individual replicates (n = 140) from two independent experiment for the droplet assay. The percentage inhibition was analyzed using one-way analysis of variance (ANOVA) and means compared with Tukey's HSD post-hoc test at α = 0.05. The correlation of antifungal efficacy of the three different analysis methods was calculated using Pearson (r) correlation analysis at *P* ≤ 0.05^60^.

## Supplementary Information

Supplementary Figures

Supplementary Video 1.

Supplementary Video 2.

Supplementary Video 3.

Supplementary Tables
