# Supplementary material for: Droplet-based microfluidics platform for antifungal analysis against filamentous fungi
Source: Sci Rep. 2021 Nov 26;11:22998. doi: 10.1038/s41598-021-02350-8 (PMC8626470; doi:10.1038/s41598-021-02350-8)
Supplement: Supplementary file 1 — Supplementary Figures [file 41598_2021_2350_MOESM1_ESM.docx]

Droplet-based microfluidics platform for high-throughput antifungal analysis against filamentous fungi

**Sehrish Iftikhar^1*^, Aurélie Vigne^1^, Julia E. Sepulveda Diaz^1^**

^1^Elvesys — Microfluidics innovation center, Elvesys, Paris, France

**Supplementary figures**
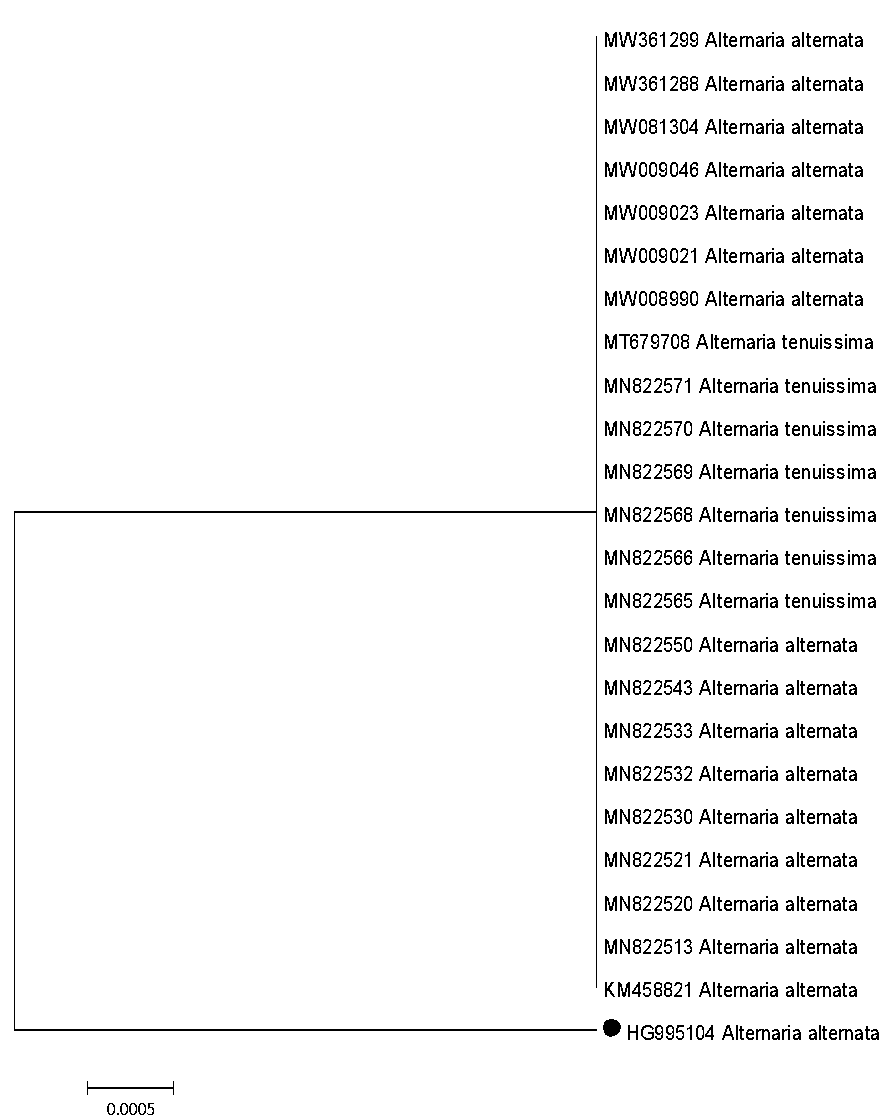


**Supplementary Figure S1:** Molecular phylogenetic analysis by maximum likelihood method of *Alternaria alternata* on the basis of ITS1 and ITS2


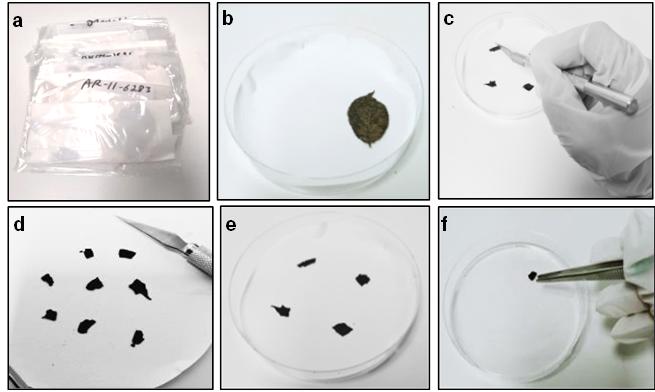


**Supplementary Figure S2:** The photograph showing the sampled plant material and isolation process of the brown spot fungus. (a) The leaves collected and packed from infected plants of potato, (b) the infected leaf with brown spot symptoms, (c) cutting the small pieces excised from the edge of lesions, (d&e) the leaf pieces washed with 1% sodium hypochlorite and sterile distilled water followed by drying on filter paper, (f) fragments placed on Petri plates containing potato dextrose agar (PDA) and incubated at 26±1 °C.

**File name: Supplementary Movie S1**

Description: The movie shows a generation of highly monodisperse droplets for single spore encapsulation with PDMS chip using droplet-based microfluidics. Oil pressure = 300 mb, spore suspension pressure = 200 mb, Scale bar = 500 μm.

**File name: Supplementary Movie S2**

Description: The movie shows a generation of highly monodispersed droplets to encapsulate a single spore of fungus with specific fungicide using a microfluidic droplet generation platform. A chip made of PC (Fluidic 719, Microfluidic ChipShop, Germany) was used. Oil pressure = 210 mb, spore suspension pressure = 200 mb, fungicide pressure = 200 mb, Scale bar = 500 μm.

**File name: Supplementary Movie S3**

Description: The movie shows generated droplets carrying the encapsulated single spore of fungus being trapped in the specific traps. Oil pressure =210 mb, spore suspension pressure = 200, fungicide pressure = 200, Scale bar = 500 μm.
