# Supplementary material for: Droplet-based microfluidics platform for antifungal analysis against filamentous fungi
Source: Sci Rep. 2021 Nov 26;11:22998. doi: 10.1038/s41598-021-02350-8 (PMC8626470; doi:10.1038/s41598-021-02350-8)
Supplement: Supplementary file 5 — Supplementary Tables [file 41598_2021_2350_MOESM5_ESM.docx]

Droplet-based microfluidics platform for high-throughput antifungal analysis against filamentous fungi

**Sehrish Iftikhar^1*^, Aurélie Vigne^1^, Julia E. Sepulveda Diaz^1^**

^1^Elvesys — Microfluidics innovation center, Elvesys, Paris, France

**Supplementary table**

**Supplementary Table S1:** The disease incidence and disease severity of brown spot disease of potato in Arras, France

| Variety | | Disease severity % | | Disease incidence % | |
| --- | --- | --- | --- | --- | --- |
| Accoustic | 38.33 | | 65.4 | |  |
| Allians | 33.18 | | 44.54 | |  |
| Aloutte | 30.81 | | 30.55 | |  |
| AR09-1825 | 45.74 | | 65.22 | |  |
| AR10-6283 | 40.31 | | 67.7 | |  |
| AR11 | 39.8 | | 65.25 | |  |
| Beyonce | 27.74 | | 72.41 | |  |
| Carolus | 17.86 | | 47.52 | |  |
| Ditta | 24.887 | | 60.57 | |  |
| Jelly | 33.66 | | 39.71 | |  |
| Jelly (Fungicide) | 22.64 | | 48.85 | |  |
| Levante | 25.61 | | 50.21 | |  |
| Levante (Fungicide) | 27.93 | | 45.77 | |  |
| Monaliza | 32.62 | | 58.46 | |  |
| Monforma (Fungicide) | 28.59 | | 62.57 | |  |
| Twinner | 66.9 | | 77.53 | |  |
| Twister | 28.78 | | 66.72 | |  |
| Twister (Fungicide) | 27.6 | | 22.5 | |  |
